# Isolation and Characterization of a Novel Pathogenesis-Related Protein Gene (*GmPRP*) with Induced Expression in Soybean (*Glycine max*) during Infection with *Phytophthora sojae*

**DOI:** 10.1371/journal.pone.0129932

**Published:** 2015-06-26

**Authors:** Liangyu Jiang, Junjiang Wu, Sujie Fan, Wenbin Li, Lidong Dong, Qun Cheng, Pengfei Xu, Shuzhen Zhang

**Affiliations:** 1 Soybean Research Institute, Key Laboratory of Soybean Biology of Chinese Education Ministry, Northeast Agricultural University, Harbin, 150030, Heilongjiang, People’s Republic of China; 2 Soybean Research Institute, Heilongjiang Academy of Agricultural Sciences, Collaborative Innovation Center of Grain Production Capacity Improvement in Heilongjiang Province, Harbin, 150086, Heilongjiang, People’s Republic of China; Agriculture and Agri-Food Canada, CANADA

## Abstract

Pathogenesis-related proteins (PR proteins) play crucial roles in the plant defense system. A novel PRP gene was isolated from highly resistant soybean infected with *Phytophthora sojae* (*P*. *sojae*) and was named *GmPRP* (GenBank accession number: KM506762). The amino acid sequences of GmPRP showed identities of 74%, 73%, 72% and 69% with PRP proteins from *Vitis vinifera*, *Populus trichocarpa*, *Citrus sinensis* and *Theobroma cacao*, respectively. Quantitative real-time reverse transcription PCR (qRT-PCR) data showed that the expression of *GmPRP* was highest in roots, followed by the stems and leaves. *GmPRP* expression was upregulated in soybean leaves infected with *P*. *sojae*. Similarly, *GmPRP* expression also responded to defense/stress signaling molecules, including salicylic acid (SA), ethylene (ET), abscisic acid (ABA) and jasmonic acid (JA). GmPRP was localized in the cell plasma membrane and cytoplasm. Recombinant GmPRP protein exhibited ribonuclease activity and significant inhibition of hyphal growth of *P*. *sojae* 1 *in vitro*. Overexpression of the *GmPRP* gene in T_2_ transgenic tobacco and T_2_ soybean plants resulted in enhanced resistance to *Phytophthora nicotianae* (*P*. *nicotianae*) and *P*. *sojae* race 1, respectively. These results indicated that the GmPRP protein played an important role in the defense of soybean against *P*. *sojae* infection.

## Introduction

Plants, being sessile, are under constant challenge by threats from an array of biological, chemical, and environmental agents. Every plant is thus forced to evolve its own structural and chemical inducible defense mechanisms for survival through various levels of challenges. These challenges activate a defense system that involves an array of induced mechanisms such as the hypersensitive response [1, 2] and the induction of PR proteins [3]. PR proteins are strongly induced in response to wounding or infection by pathogens, accumulate abundantly at the site of infection, and contribute to systemic acquired resistance (SAR) [4, 5].

The production and accumulation of PR proteins in plants in response to invading pathogens or related abiotic stresses are among the crucial steps in the inducible portion of a plant’s self-defense mechanism [6]. Many PR proteins have been characterized in recent years, and they are classified into 17 families [7, 8]. Some PR proteins have been characterized as chitinases, β-1,3-glucanases [9], ribonucleases [10–13], thaumatin-like proteins (TLPs) [14–16], proteinase inhibitors (PIs) [17, 18], plant defensins (PDFs) [19], and lipid transfer proteins (LTPs) [20].

PR proteins not only accumulate in various parts of normal tissues, but are induced by pathogen infection and improve the defensive capacity of plants [21]. The induction of *PR10* gene expression has been demonstrated in various plant species following infection by pathogens [22–24]. PR2 and PR3 are strongly induced when plants respond to wounding or infection by fungal, bacterial, or viral pathogens [25, 26]. The PR6 family can be induced upon inoculation with *Phytophthora infestans* and *Pseudomonas syringae* pv. Tomato [27, 28]. The PR5 proteins appear to be mainly involved in plant defensive systems that counteract infection by pathogens [15, 29]. PR4 and PR1 proteins have been reported to have antifungal activity and resistance-related properties in many plant species [10, 30].

To further understand the function of *PR* genes in plant defense reactions, the expression patterns of *PR* genes was analyzed by various stimulus. *PR* expression is known to be regulated by signaling compounds such as ABA, ET, JA, and SA [31–35]. *PR* genes have been shown to be induced by various abiotic stresses, such as treatments with NaCl, heat, cold, PEG [31], UV irradiation [36], and ozone [37]. Some PR proteins have also been reported to accumulate under specific physiological conditions, such as pollen development [38], leaf senescence, fruit development and ripening [39–41].

Plants activate the expression of different *PR* genes in response to pathogens to improve the defensive capacity of plants. There are also several reports on overexpressing *PR* genes, resulting in enhanced tolerance to pathogen infection [42–44]. For example, overexpression of *PR5* genes has been shown to enhance resistance to *Rhizoctonia solani* and *Phytophthora infestans* [45, 46], and overexpression of *CABPR1* in tobacco plants enhances tolerance not only to heavy metal stresses but also to pathogen attack [47].

Although the biological and biochemical functions of PR proteins have been studied for several decades, the molecular mechanisms of many PR proteins remain unknown [8, 48, 49]. Thus far, PR proteins have been identified in numerous plants, including hot pepper [12], corn [13], potato [50], wheat [51], lily [52], rice [53], and soybean [54]. However, some PR proteins were not grouped into the 17 PR protein families and were simply named pathogenesis-related proteins (PRPs). Phytophthora root and stem rot of soybean (*Glycine max* (L.) Merr.), caused by *Phytophthora sojae* Kaufmann and Gerdemann, is a destructive disease throughout the soybean-growing regions worldwide [55]. We previously reported, using SSH and cDNA microarrays, that a highly upregulated *PRP* gene (termed *GmPRP*) was induced by *P*. *sojae* in the highly resistant soybean cultivar ‘Suinong 10’ [56]. However, no further studies have been conducted to examine the expression, localization and biochemical activity of this PRP. The objective of the present study was to conduct a functional analysis of *GmPRP* in the defense against *P*. *sojae* for its possible use as a new tool for the management of Phytophthora root and stem rot in soybean.

## Materials and Methods

### Plant materials and pathogen inoculation

This study used ‘Suinong 10’, a popular soybean cultivar with high genetic resistance against the predominant *P*. *sojae* race 1 in Heilongjiang, China [57]. Seeds of ‘Suinong 10’ were planted in pots filled with sterile vermiculite under a 14-h photoperiod at a light intensity of 350 molm^-2^ s^-1^ at 25°C and 10-h darkness at 18°C in a growth chamber. Ten days after planting, seedlings at the first-node stage (V1) [58] were used for various treatments.

For *P*. *sojae* treatment, the soybean plants were inoculated with *P*. *sojae* zoospores following the methods described by Ward *et al*. (1979) [59] and Morris *et al*. (1991) [60] with minor modifications. Zoospores were developed using the procedure of Ward *et al*. (1979) [59], and the concentration was estimated using a hemocytometer to approximately 1 × 10^5^ spores mL^-1^. All of the seedlings were incubated in a mist chamber at 25°C, with 100% relative humidity and a 14-h photoperiod at a light intensity of 350 μmolm^-2^ s^-1^. The unifoliolate leaves of inoculated ‘Suinong 10’ were harvested at 0, 3, 6, 12, 24, 36, and 72 h after the treatment, immediately frozen in liquid nitrogen, and kept at -80°C until quantitative RT-PCR analysis.

### RNA extraction and cDNA analysis

Total RNA was extracted from the leaves of the *P*. *sojae*-inoculated soybean plants using a Trizol kit (Invitrogen, Shanghai, China) according to the manufacturer’s instructions. The quality and concentration of the RNA samples were examined by agarose gel electrophoresis and analyzed using a Lambda 35 UV/vis Spectrometer (Perkin Elmer, Massachusetts, USA). Total RNAs were converted into cDNAs using a random oligo dT primer and M-MLV reverse transcriptase according to the manufacturer’s instructions.

### Cloning of a novel *GmPRP* gene

The first-strand reaction product from the obtained cDNA was used to clone the full-length *GmPRP*. Rapid amplification of cDNA ends (RACE) was conducted from soybean mRNA using the CLONTECH SMART RACE cDNA Amplification Kit (Clontech, USA). The gene-specific primers, *GmPRP* (F: TGATGACGCCATCTTTAGTACC; R: CGGCTAGAGCAGCACAAGTTCAA), were used to amplify *GmPRP* gene. The PCRs were performed under the following conditions: 94°C for 3 min, then 30 cycles at 94°C for 30 s, 57°C for 30 s, and 72°C for 30 s, with a final extension at 72°C for 10 min. The PCR products were ligated into the pGM-T Easy vector. The clones containing approximately 600-bp and 250-bp fragments were identified by DNA sequencing. Sequence alignment was conducted using DNAMAN software. A clone containing a fragment of approximately 925 bp was obtained and sequenced.

Protein sequence similarity analysis was performed using the BLAST algorithm (http://www.ncbi.nlm.nih.gov/blast). The programs ClustalW (http://www.ebi.ac.uk/clustalw/) and Multiple Alignment ShowB (http://www.bio-soft.net/sms/index.html) were used for multiple sequence alignment. The potential phosphorylation sites of GmPRP protein were analyzed using NetPhos software (http://www.cbs.dtu.dk/services/NetPhos). The phylogenetic relationships of *GmPRP* homologs in plants were constructed using the neighbor-joining method with the program MEGA 5.1. The structural features of GmPRP protein were analyzed using the Expert Protein Analysis System (http://www.expasy.org/). The promoter sequences of *GmPRP* gene upstream of ATG were analyzed using Plantcare software (http://bioinformatics.psb.ugent.be/webtools/Plantc-are/html/).

### Quantitative Real-time PCR analysis

Quantitative Real-time PCR (qRT-PCR) was conducted to examine the expression of *GmPRP* under abiotic (phytohormone and chemical) and biotic (*P*. *sojae*) stresses in ‘Suinong 10’ soybean. For phytohormone and chemical treatments, ‘Suinong 10’ soybean leaves were sprayed with 0.5 mM SA, 50 mM ABA, or 100 mM JA and harvested for RNA isolation at 1, 3, 6, 9, 12, and 24 h after the treatments and were subjected to qRT-PCR analysis. For treatment with ET, ‘Suinong 10’ soybean plants were kept in a chamber equilibrated with 5% (v/v) gaseous ethylene. Control experiments were carried out in an identical chamber without ethylene.

The leaves of ‘Suinong 10’ soybean seedlings were inoculated with zoospores of *P*. *sojae* race 1 following the methods described by Ward et al. (1979) [59] and Morris et al. (1991) [60] with minor modifications and harvested at 6, 12, 24, 36, 48, and 72 h after the treatment for qRT-PCR analysis. Two specific primers, *GmPRP* F: TTCAGCCTAAACGGAAGGAAGCCT and *GmPRP* R: TTGTCGTGAAGGCCTTATGGGATG), were used to determine the expression level of *GmPRP*. qRT-PCR amplifications were performed using a real-time RT-PCR kit according to the manufacturer’s instructions (Takara, Japan) on the CFX96 TouchTM Real-Time PCR Detection System (BioRad, USA). One microgram of total RNA was used for each reverse transcription. Each amplification reaction was performed with 1 μL of the resultant first-strand cDNA solution, 0.2 μM of each primer and 2×SYBR Green PCR Master Mix, in a total reaction volume of 20 μL. The PCR cycling conditions were as follows: 95°C for 5 s, 60°C for 20 s, 72°C for 20 s for 40 cycles and 60°C for 1 min. The relative levels of *GmPRP* mRNA were evaluated against the soybean housekeeping gene *GmActin 4* (GenBank accession number: AF049106) amplified with specific primer pairs (F: CTTGGAGGATCATGTTCGGTT; R: GCATCACAGT-GCAATCTAGCT). For tissue distribution analysis, the tissue with the lowest expression level was used as calibrator. The relative expression of target gene in different tissues of soybean or in different transgenic lines of tobacco and soybean was calculated using the 2^−∆∆CT^ method, and each assay was conducted with three technological replications and statistically analyzed using Student’s t-test (*P < 0.05, **P < 0.01). Bars indicate the standard error of the mean (SE).

### Subcellular localization of the *GmPRP* protein in onion epidermal cells

To analyzed the subcellular localization of the GmPRP protein, the coding sequences of Gm*PRP* were first amplified using primer pairs for *GmPRP*-GFP (F: CCCATGGCGTCATCAAGTGT; R: GGACTAGTGCCGGTGTTCCTGAGTAC; the underlined bases are for the restriction sites *Nco*I and *Spe*I, respectively). After digestion with *Nco*I and *Spe*I, the PCR products was ligated into pCAMBIA1302 vector to produce the recombinant plasmid *CaMV35S-GmPRP*-GFP. The recombinant plasmid was transformed into the Arabidopsis protoplast cells using the method as described by Yoo et al. (2007) [61]. The vector *CaMV35S*-GFP was used as a control. After 20 h, GFP fluorescence was observed with a Leica TCSSP2 fluorescent stereomicroscope (Leica Microsystems, Wetzlar, Germany).

### Expression, purification and renaturation of the *GmPRP* protein

The coding region of the *GmPRP* gene were amplified using specific primers, *GmPRP* (F: GGAAGATCTTATGGCGTCATCAAGTGT, with the *Bgl*II site underlined; R: CCGCTCGAGG-CCGGTGTTCCTGAGTAC-3, with the *Xho*I site underlined). The PCR products were digested with *Bgl*II and *Xho*I and was ligated into the pET-29b vector. The recombinant plasmid pET29b-*GmPRP* was transformed into BL21 (DE3) cells for protein expression. BL21 (DE3) strains transformed with the pET29b-*GmPRP* plasmid were grown in LB medium containing 50 mg/mL kanamycin at 37°C to an absorbance of 0.5 at 600 nm. The cultures were induced by 0.5 mM IPTG. After 4 h of induction, the cells were harvested by centrifugation at 4000 rpm for 10 min at 4°C. The expression and purification of the recombinant protein were performed as previously described by Xu et al. (2014) [50].

### Antimicrobial activity assays for recombinant *GmPRP* protein

The inhibition of the growth of *P*. *sojae* by the GmPRP protein was assayed using the method described by Schlumbaum et al. (1986) [62]. *P*. *sojae* race 1 was grown on carrot agar plates at 28°C. The sterile filter paper discs were treated with 15 μg, 25 μg, or 35 μg of the renatured recombinant GmPRP protein, and treatment with 35 μg of boiled recombinant GmPRP protein served as the control. The growth zones of the pathogen were observed and photographed using a Canon IXUS 860IS camera after incubation at 28°C for 24 h.

### Ribonuclease activity assays with recombinant *GmPRP* protein

To elucidate the ribonuclease activity of the GmPRP protein, RNase activity assays were performed according to the method described by Bantignies et al. (2000) [63] with minor modifications. Briefly, 2 μg, 4 μg, or 6 μg of purified recombinant protein and 10 μg of total RNA extracted from ‘Suinong 10’ soybean leaves were mixed in separate 20 μL reaction mixtures and incubated at 37°C for 2 h. The amount of solubilized RNA in the supernatant was determined by UV absorbance at 260 nm (OD_260_) with a NanoDrop ND-1000 spectrophotometer (Thermo). The water containing only dissolved RNA was used as a negative control.

### Vector construction and tobacco, soybean transformation

*GmPRP* was amplified using the pGEM-T easy vector template and gene-specific primers. Two specific primers for *GmPRP* (F: GGGGGATCCAAAGATGGCGTCATC, with the *BamH*Ⅰ site underlined; R: ACAAGCCAGAGCTCCAACAACTGCAAT, with the *Sac*Ⅰ site underlined) were used to amplify the coding region of the *GmPRP* gene. The PCR products were cloned into the pGM-T easy vector followed by transformation into *E*. *coli* DH5α cells (Shanghai Biotech Inc., Shanghai, China) and sequenced. The PCR products and pCAMBIA3301 (www.camia.org), using the *bar* gene as the selective marker and *GUS* as the reporter gene, were digested with *BamH*Ⅰ and *Sac*Ⅰ. The recombinant plasmid pCAMBIA3301-*GmPRP* was produced by ligating the digested PCR products with the digested pCAMBIA3301 plasmid. *E*. *coli* DH5α cells were transformed using the pCAMBIA3301-*GmPRP* plasmid. The plant expression vector was introduced into *Agrobacterium tumefaciens* LBA4404 by the freezing and thawing method. ‘Dongnong 50’ soybean, which is susceptible to *P*. *sojae* race 1, and ‘Havana 425’ tobacco, which is susceptible to *Phytophthora nicotianae* Breda, were used for gene transformation experiments. Professor WX Shan of the College of Plant Protection, Northwest Agriculture and Forestry University, China kindly provided the *P*. *nicotianae* isolate. The tobacco leaf discs were transformed according to the methods described by Horsch et al. (1985) [64], and the soybean cotyledonary nodes were used as explants transformed with the *Agrobacterium*-mediated method described by Wang et al. (2008) [65]. The T_1_ seeds were collected, dried at 25°C, and grown in soil to test the transgenic tobacco and soybean plants. T_2_ seeds were set the same way as T_1_ seeds.

### PCR analysis of transgenic plants

To confirm transgene insertion into the transgenic tobacco (T_2_) and soybean plants (T_2_), genomic DNA was extracted by the CTAB method, and PCR analysis was conducted. Two primers (F: ATATCCGAGCGCCTCGTGCAT, R: GGTCTGCACCATCGTCAACCACT) were designed to target the regions of the *bar* reporter gene. Using genomic DNA as the template, PCR was performed with a pre-denaturing condition of 94°C for 3 min, followed by 30 cycles at 94°C for 30 s, 68°C for 30 s, 72°C for 30 s, and finally 72°C for 8 min.

### Southern hybridization analysis

Southern hybridization analysis of PCR-positive soybean plants (T_2_) was performed using the DIG High Prime DNA Labeling and Detection Starter Kit II (Roche Cat., Germany) according to the manufacturer’s instructions. Specifically, 20μg of genomic DNA was digested with *Hind*III and electrophoresed on a 1.0% agarose gel. The DNA was transferred to nylon membranes (Schleicher and Schuell, Keene, NH) using the alkaline transfer protocol and UV cross-linking [66]. The PCR products of the bar genes and the target gene from the plasmid were used as the probes, which were labeled using digoxigenin (DIG)-11-dUTP with DIG High Prime DNA Labeling reagents (Roche, Mannheim, Germany). Hybridization was conducted at 42–45°C, and washing, blocking, and detection were performed according to the manufacturer’s instructions. The Southern hybridization was then exposed to X-ray film (Kodak, Japan) using two intensifying screens at room temperature for 20 min and subsequently developed.

### Resistance identification of transgenic tobacco and soybean plants

To investigate whether the *GmPRP* gene could enhance resistance, inoculum preparation and artificial inoculation procedures were modified from those described by Dou et al. (2003) [67]. The leaves of transgenic tobacco plants that were tested through qRT-PCR were inoculated with a *P*. *nicotianae* Breda inoculum, and those of the soybean plants that were tested through qRT-PCR and Southern analysis were inoculated with a *P*. *sojae* race 1 inoculum. The leaves were incubated for 72 h in a mist chamber at 25°C and 90% relative humidity under a 14-h photoperiod with light intensity of 350 molm^-2^ s^-1^. Non-transgenic leaves were used as controls. The disease symptoms on each leaf were observed and photographed using a Canon IXUS 860IS camera at 24, 48 and 72h after the inoculation.

## Results

### Cloning and sequencing of *GmPRP* cDNA

**Fig 1:**
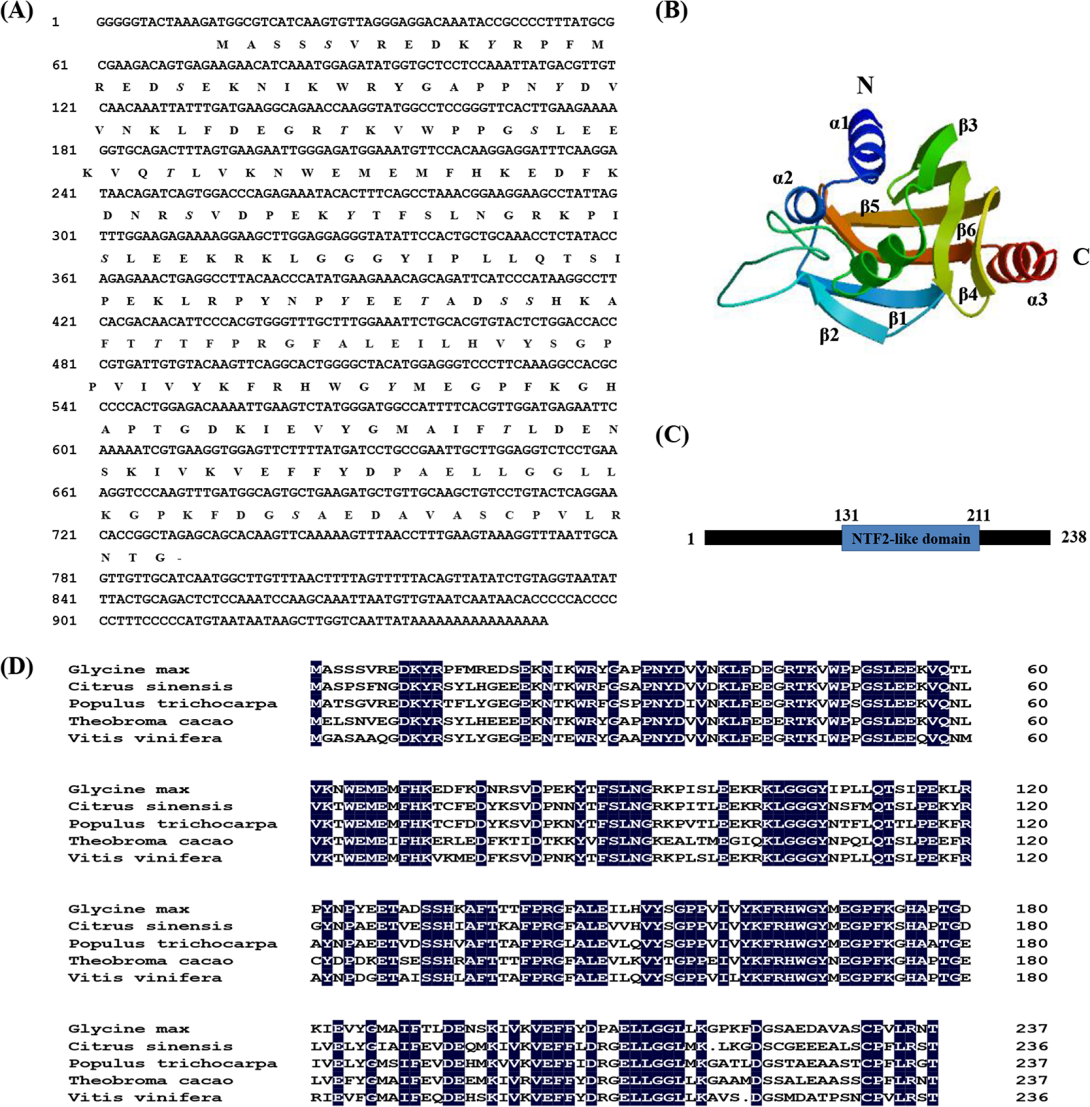
Nucleotide and amino acid sequences of *GmPRP* cDNA. (A) Nucleotide and amino acid sequences of *GmPRP* cDNA. Putative phosphorylation sites are marked in bold italics. (B) Analysis of the 3D structure and conserved motifs of GmPRP. The 3D structure of GmPRP. The N-terminal, C-terminal, α-helix, and β-strand are marked in bold italics. (C) The conserved motif of the GmPRP protein. The predicted GmPRP protein contained a conserved motif at residues 131–211 aa that belonged to the NTF2-like superfamily. (D) Alignments of the amino acid sequences of the GmPRP with other plant PRP proteins. Comparison of the predicted amino acid sequence of GmPRP with other plant PRPs from *Vitis vinifera* (XP002262980), *Populus trichocarpa* (XP002306682), *Citrus sinensis* (XP006472936) and *Theobroma cacao* (XP007019416). Conserved residues are shaded in black.

The full-length cDNA sequence was obtained from ‘Suinong 10’ soybean using RACE. The clone, designated *GmPRP* (GenBank accession no. KM506762), was chosen for further functional analysis. It comprised 952 bp with a single open reading frame (ORF) of 717 nucleotides, encoding a polypeptide of 238 amino acids with a calculated molecular mass of 27.225 kDa and a theoretical PI of 7.07. The nucleotide sequence also showed a 5’ untranslated region (5’ UTR) of 13 nucleotides and a 3’ UTR of 222 nucleotides along with a poly-A signal (AAAAAAAAAAAAAAAA) at 936–952 bp. A database search (http://www.cbs.dtu.dk/services/signalp/) indicated that GmPRP contained no signal peptide. Searches of the NCBI and Phytozome databases (http://www.n.nihcbi.nlm.gov/; http://www.phytozome.net/soybean) revealed that the *GmPRP* gene had a 694-bp intron and was located on chromosome 6 with two copies. The software NetPhos (http://www.cbs.dtu.dk/services/NetPhos) predicted that GmPRP had eight serines (Ser 5, 19, 52, 79, 96, 131, 132, 223, in bold italics), five tyrosines (Tyr 11, 33, 85, 125, 167, in bold italics), and five lysines (Lys 45, 59, 128, 138, 191, in bold italics) as potential phosphorylation sites (Fig 1A). The 3D structure of the GmPRP protein consisted of a 10-amino-acid C-terminal α-helix (α3) surrounded by a six-stranded antiparallel β-sheet (from β1 to β6) and three N-terminal α-helices (α1, α2 and α3), which are two short α-helices and a long α-helix between the β2 and β3 sheets. The connection sequences between the α-helix and β-strand were short loop structures (Fig 1B). The predicted GmPRP protein contained a conserved motif at residues 131–211 aa that belonged to the NTF2-like superfamily (Fig 1C).

**Fig 2:**
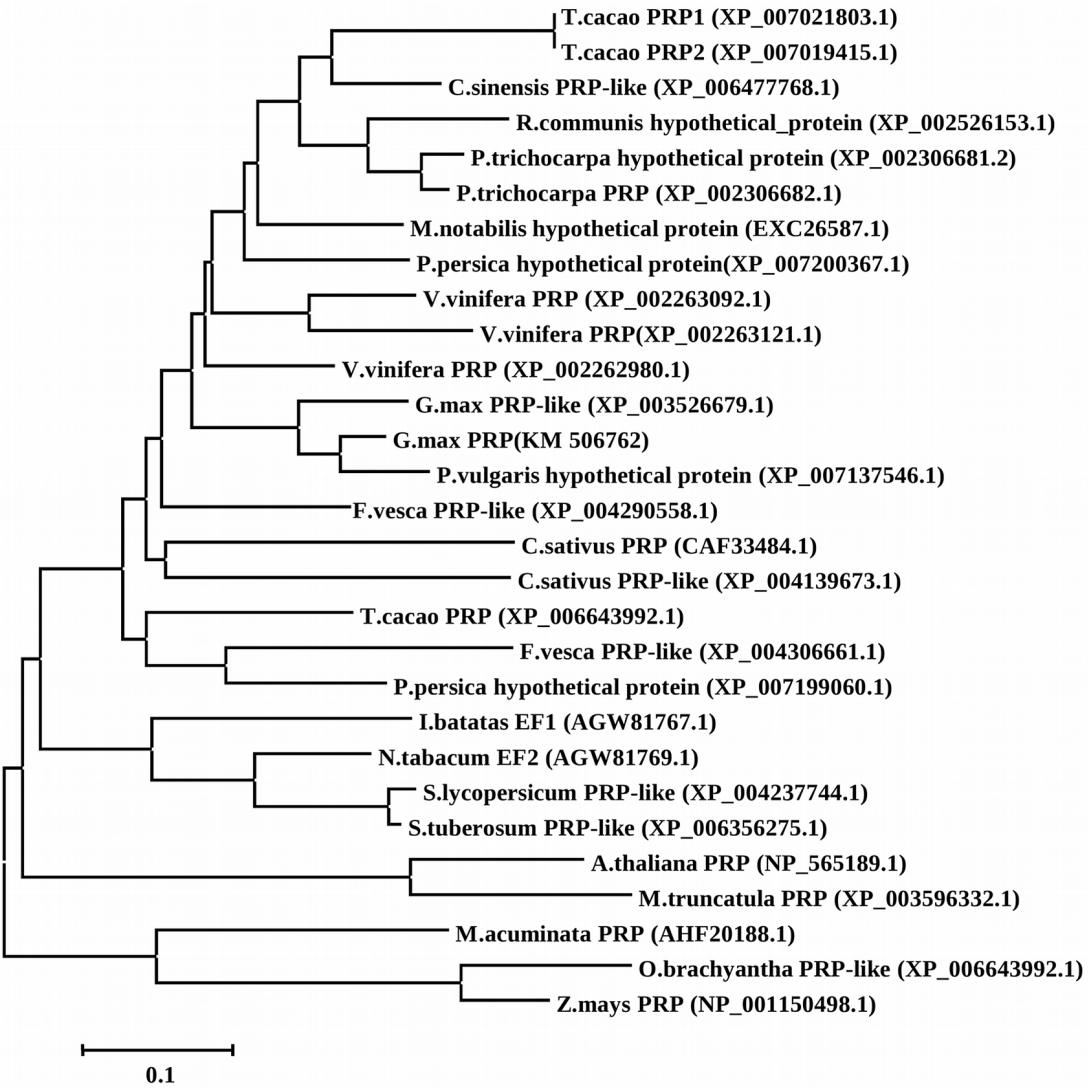
Phylogenetic relationships of GmPRP homologs in plants, constructed using the program MEGA 5.1.

Sequence comparison showed that the putative GmPRP protein was homologous to PRPs from other plants. Its deduced amino acid sequence showed 74%, 73%, 72% and 69% similarity to *Vitis vinifera* (XP002262980), *Populus trichocarpa* (XP002306682), *Citrus sinensis* (XP006472936) and *Theobroma cacao* (XP007019416), respectively (Fig 1D). The phylogenetic relationships of GmPRP homologs in plants were constructed using the neighbor-joining method with the program MEGA 5.1. A neighbor-joining (NJ) phylogram was used to construct a phylogenetic tree based on the deduced sequence of GmPRP with other members of the PRP family, indicating that they may share a common ancestor and perform similar functions (Fig 2).

### Differential transcript levels of *GmPRP* in response to abiotic stresses

The responsiveness of a gene to certain stresses may indicate its defensive roles through several mechanisms. Therefore, the expression patterns of the *GmPRP* gene under various abiotic stresses and stress-related chemicals, including SA (0.5 mM), ABA (50μM), JA (100μM), ET (5% (v/v)) and *P*. *sojae* race 1, were investigated using qRT-PCR. The time-course qRT-PCR data were analyzed to examine the effects of abiotic stress conditions on *GmPRP* gene expression.

**Fig 3:**
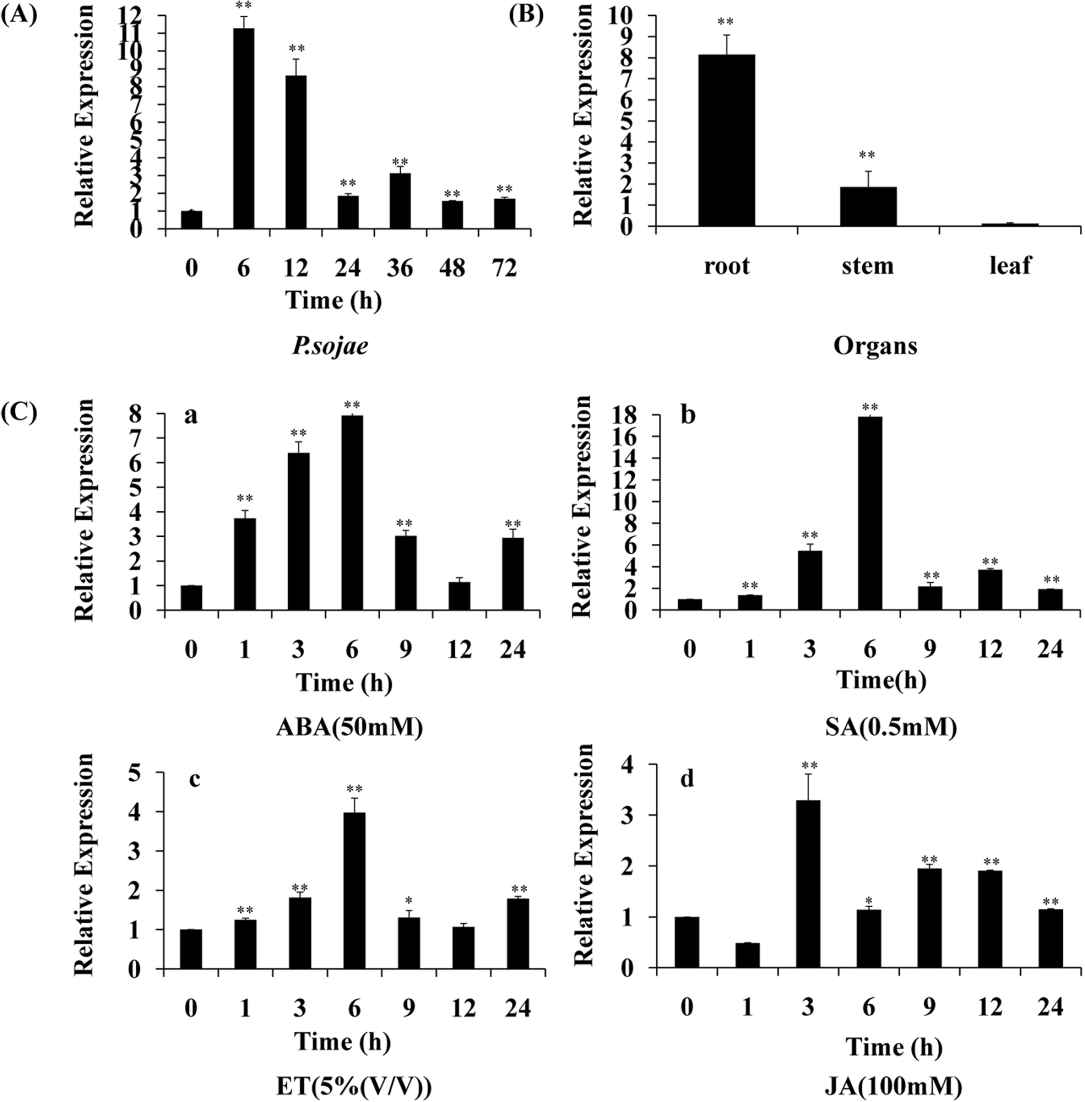
Relative expression of *GmPRP* mRNA in leaves of soybean plants after abiotic or biotic stress. (A) Infection study. The leaves of ‘Suinong 10’ soybean seedlings inoculated with zoospores of P. sojae race 1 were also harvested at 6, 12, 24, 36, 48, and 72 h. (B) Tissue-specific expression study. *GmPRP* expression in the organs of sterile seedlings. (C) Hormone study. The leaves were collected from soybean plants at 1, 3, 6, 9, 12, and 24 h after treatment with ABA (50 mM) (a), SA (0.5 mM) (b), ET (5%(v/v)) (c), or JA (100 mM) (d). The experiments were performed at least three times for each treatment. Data shown are representative results. Statistical analyses were performed using Student’s t-test (*P < 0.05, **P < 0.01). Bars indicate the standard error of the mean (SE).

The transcripts of the *GmPRP* rapidly increased in leaf under *P*. *sojae* infection, reaching a maximum level at 6 h after the treatments, followed by a rapid decline (Fig 3A). *GmPRP* was constitutively expressed, with the highest expression in roots, followed by the stems and leaves (Fig 3B). Under ABA, SA and ET treatments, *GmPRP* mRNA transcripts accumulated in leaf and reached maximum levels at 6 h, followed by a decline (Fig 3C-a–3C-c). JA treatment induced the downregulation of *GmPRP* transcripts at the beginning of the treatment, and the transcripts remained at a low level at 1 h. However, *GmPRP* transcripts increased and reached a maximum level at 3 h after the treatments, followed by a decline (Fig 3C and 3D).

### Subcellular localization of the *GmPRP* protein

**Fig 4:**
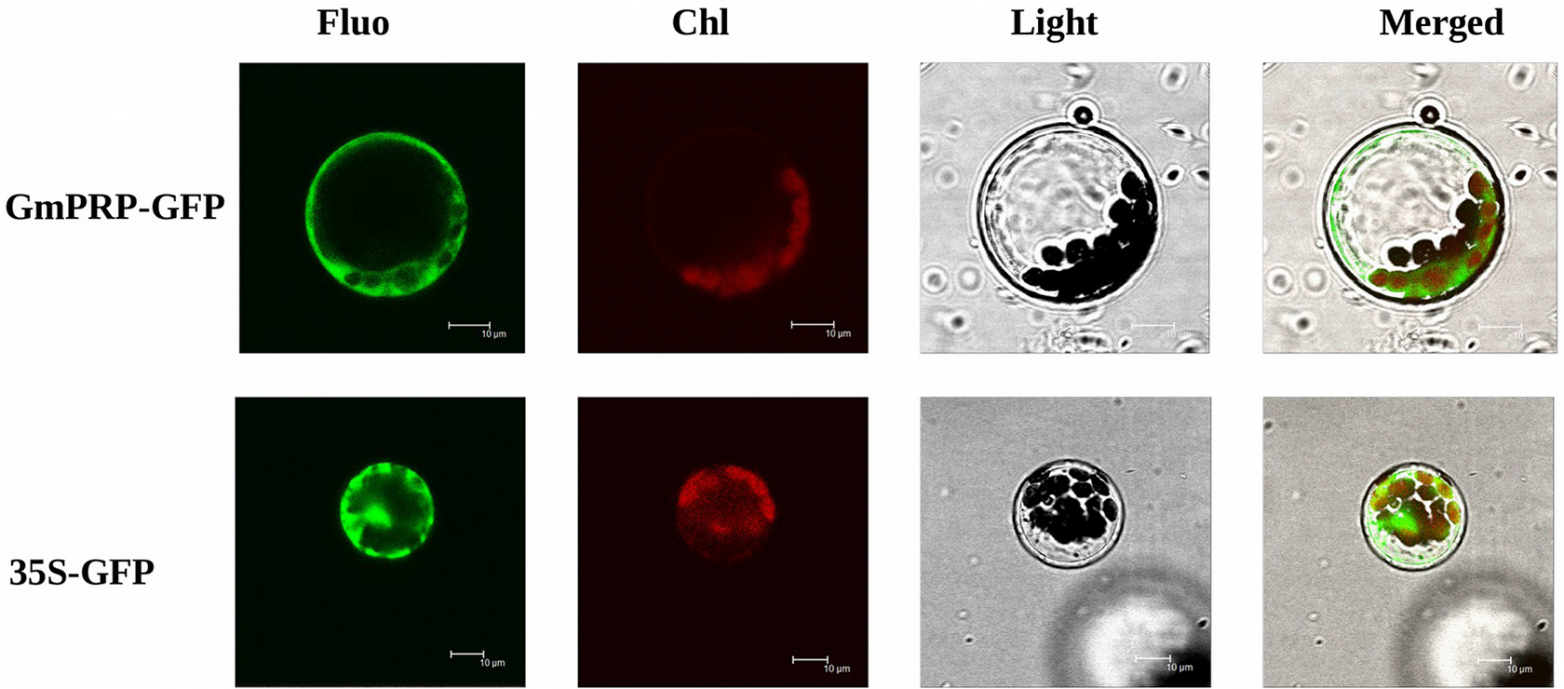
Subcellular localization of the *GmPRP*-GFP fusion protein. Arabidopsis protoplast cells expressing either *GmPRP*-GFP fusion protein (top) or GFP (bottom) were observed by fluorescence stereomicroscopy. Scale bar = 100 μm.

To investigate the subcellular localization of the GmPRP protein, the *GmPRP*-GFP fusion gene was transformed into the Arabidopsis protoplast cells. As depicted in Fig 4, the GFP signal of the control was present in both cytoplasm and nuclear, the GFP signal of the *GmPRP*-*GFP* fusion gene was present in the cell plasma membrane and cytoplasm.

### Expression of *GmPRP* in E. coli and properties of the *GmPRP* protein

Without the induction by 0.5 mM IPTG, all extracts of *E*. *coli* with or without the pET-29b vector produced negative results. However, the protein expression was remarkably enhanced by IPTG and increased from 2 to 6 h. Maximum expression of the protein was achieved at 4 h (S1A Fig). The purified recombinant protein migrated at 30 kDa in SDS-PAGE (S1B Fig). That value was consistent with the predicted molecular weight calculated from the amino acid sequence.

### Antimicrobial activity assays of recombinant *GmPRP* protein

**Fig 5:**
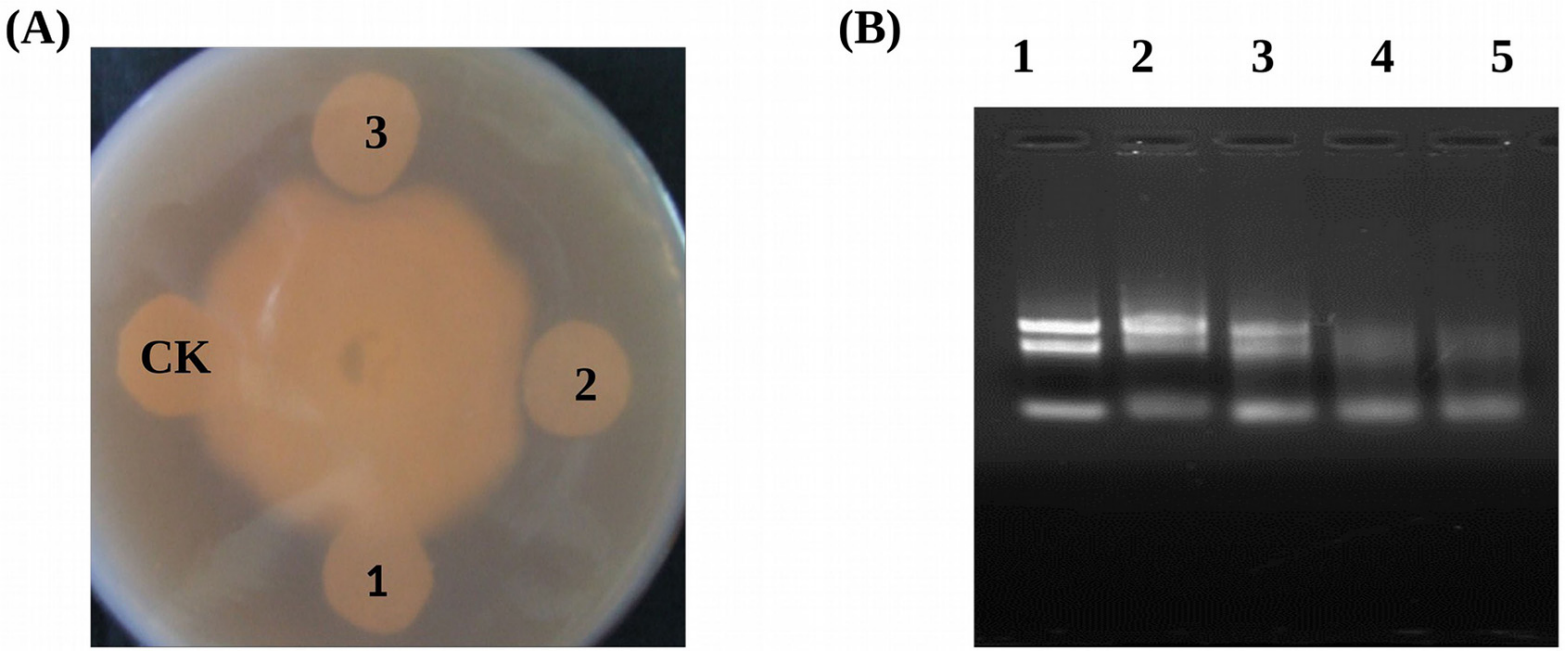
Antimicrobial activity ribonuclease activity and assays for the recombinant GmPRP protein. (A) Inhibition of *P*. *sojae* race 1 growth by purified recombinant GmPRP. 1, 15 μg renatured recombinant GmPRP protein; 2, 25 μg renatured recombinant GmPRP protein; 3, 35μg renatured recombinant GmPRP protein; CK, 35 μg boiled renatured recombinant GmPRP protein. (B) Ribonuclease activities of recombinant GmPRP proteins on soybean RNA. Gel electrophoresis using 1.0% agarose gel was performed to separate hydrolyzed RNAs. Each reaction mixture containing total RNA from soybean was incubated for 2 h at 37°C. Lane 1, 5 μg RNA+ Elution buffer; Lane 2, 5 μg RNA+ 6 μg of boiled dialytically renatured GmPRP protein. Lane 3, 5 μg RNA+ 2 μg dialytically renatured GmPRP protein; Lane 4, 5 μg RNA+ 4 μg dialytically renatured GmPRP protein; Lane 5, 5 μg RNA+ 6 μg dialytically renatured GmPRP protein.

To examine the antimicrobial activity effect of the recombinant GmPRP protein on the growth of *P*. *sojae* 1, filter-paper discs containing recombinant GmPR10 protein (15, 25, 35 μg) were placed near the front of the growing hyphae of *P*. *sojae* 1. After incubation, a 2- to 3-mm zone with inhibited hyphal growth was detected when 25 μg of recombinant protein was applied to the filter-paper discs, and the bacteriostatic effect was enhanced by the application of 35 μg of the protein (Fig 5A). A control filter-paper disc containing boiled recombinant protein did not show an inhibitory effect.

### Ribonuclease activity assays of recombinant *GmPRP* proteins

The total RNA isolated from the leaves of ‘Suinong 10’ soybean was incubated with or without recombinant GmPRP protein (Fig 5B). The elution buffer did not degrade the total RNA (Fig 5B, lane 1). The control sample, which was incubated with the boiled dialytically renatured GmPRP protein, did not show significant RNase activity against soybean RNA (Fig 5B, lane 2). However, when incubated with the non-boiled dialytically renatured GmPRP protein, RNase activity was clearly visible through the migration of the degradation products in the agarose gel (Fig 5B, lane 3, 4, 5).

### Detection of transgenic tobacco and soybean

**Fig 6:**
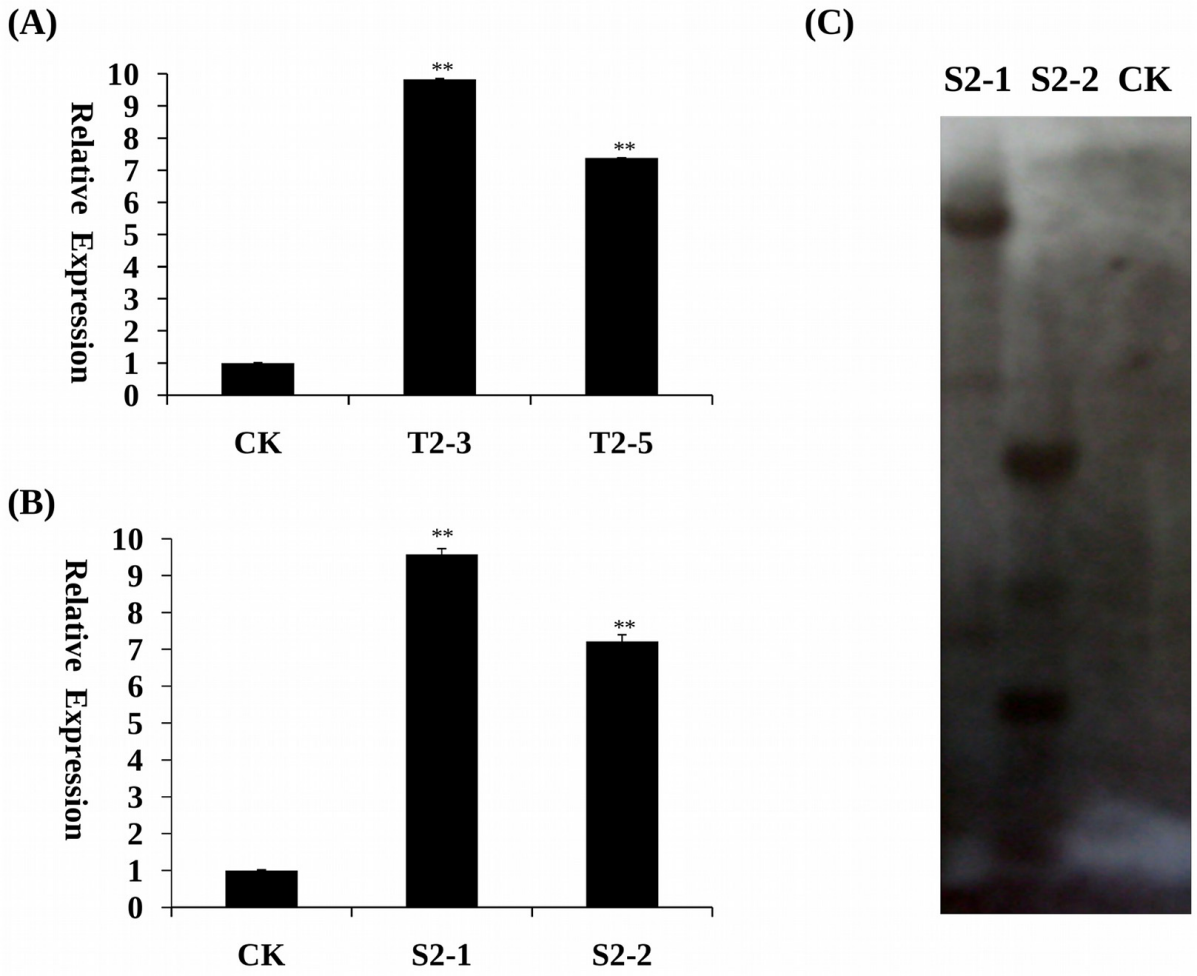
Expression of *GmPRP* gene in the leaves of transgenic tobacco and soybean plants. (A) qRT-PCR determining the relative bundance of *GmPRP* (line T2-3 and T2-5) in the transgenic tobacco plants. The non-transgenic tobacco plants only were used as a control. (B) qRT-PCR determining the relative bundance of *GmPRP* (line T2-3 and T2-5) in the transgenic soybean plants. The non-transgenic soybean plants only were used as a control. (C) Genomic Southern hybridization analysis confirming stable integration and expression of *GmPRP* in young fully expanded leaves of transgenic soybean plants (CK, wild-type untransformed soybean control, line S2-1 and S2-2 independently transformed T2 transgenic events). All data represent the mean values of three replications.

To confirm transgene insertion in transgenic tobacco (T_2_) and soybean plants (T_2_), the CTAB method was used to extract genomic DNA from young, fully expanded leaves. Finally, 10 independently transformed T_2_ transgenic tobacco plants (numbered from T2-1 to T2-10) and 6 independently transformed T_2_ transgenic soybean plants (numbered from S2-1 to S2-6) were identified by PCR. qRT-PCR of two tobacco transgenic lines (T2-3, T2-5) containing *GmPRP* and two soybean transgenic lines (S2-1, S2-2) containing *GmPRP* showed that *GmPRP* expression was significantly higher than non-transgenic tobacco and soybean plants (Fig 6A and 6B). To determine the copy number of *GmPRP* in the genome of T_2_ transgenic soybean, Southern hybridization analysis was performed. Two independently transformed T_2_ transgenic soybean plants (S2-1 and S2-2) were detected to have three and four copies, respectively (Fig 6C). These results suggest that the *GmPRP* gene was transformed successfully into soybean plants.

### Enhanced resistance to *P*. *nicotianae* and *P*. *sojae* in transgenic plants

**Fig 7:**
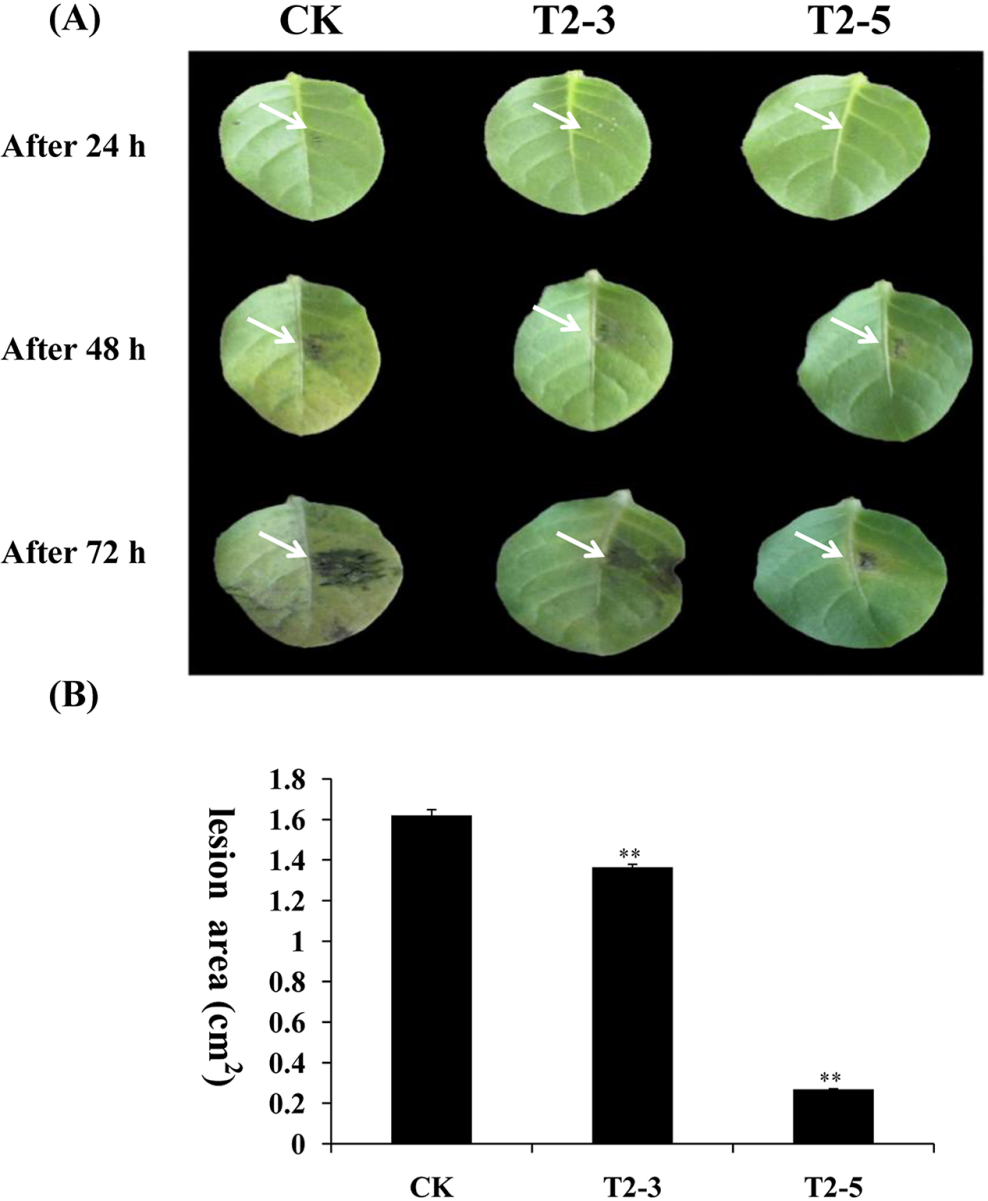
Overexpression of *GmPRP* in tobacco leaves enhanced the resistance to *P*. *nicotianae*. (A) Disease symptoms after infection with *P*. *nicotianae*. Row a, tobacco leaves 24 h after inoculation. Row b, tobacco leaves 48 h after inoculation. Row c, tobacco leaves 96 h after inoculation. Column CK, leaves of non-transgenic tobacco. Columns T2-3 and T2-5, leaves of transgenic tobacco. (B) Lesion size of transgenic tobacco leaves infection with *P*. *nicotianae* after 96 h. All data represent the mean values of three replications.

**Fig 8:**
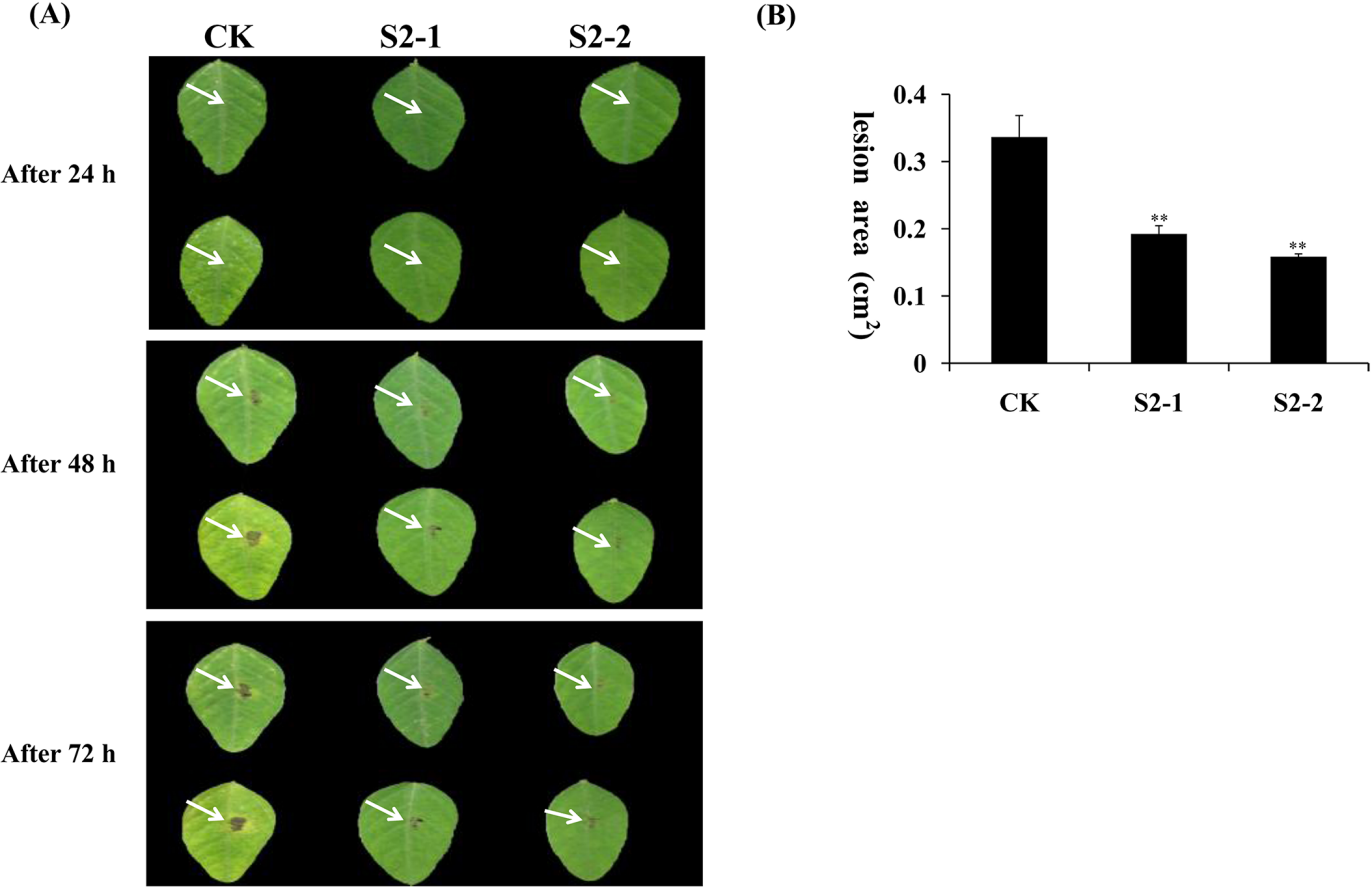
Overexpression of the *GmPRP* gene in soybean leaves enhanced the resistance to *P*. *sojae*. (A) Disease symptoms after infection with *P*. *sojae*. (a) Soybean leaves 24 h after inoculation. (b) Soybean leaves 48 h after inoculation. (c) Soybean leaves 96 h after inoculation. Column CK, leaves of non-transgenic soybean. Columns S2-1 and S2-2, leaves of transgenic soybean. (B) Lesion size of transgenic soybean leaves infection with *P*. *sojae* after 96 h. All data represent the mean values of three replications.

*GmPRP* was overexpressed in tobacco and soybean to evaluate the antimicrobial activity of this protein *in vivo*. Two transgenic tobacco plants (T2-3 and T2-5) and two transgenic soybean plants (numbered S2-1 and S2-2) were selected to investigate the susceptibility or resistance to *P*. *nicotianae* and *P*. *sojae* race 1, respectively. After 72 h of incubation with *P*. *nicotianae* or *P*. *sojae* race 1, remarkable differences in the development of disease symptoms were observed between the transgenic and non-transgenic tobacco and soybean plants. After 72 h of incubation with *P*. *nicotianae*, severe symptoms (necrosis and chlorosis) around the infection areas were observed in non-transgenic tobacco plants (Fig 7A, Lane CK), but the transgenic tobacco plants showed only slight lesions (Fig 7A, Lane T2-3, T2-5). The lesion area of the inoculated CK (1.62 cm^2^) is significantly different from the lesion area of transgenic lines T2-3 and T2-5 (only 1.36 and 0.27 cm^2^, respectively) (Fig 7B). After 72 h of incubation with *P*. *sojae* race 1, the leaves of the non-transgenic soybean plants exhibited clear and large water-soaked lesions compared with those of the transgenic plants (Fig 8A). The lesion area of the inoculated CK (0.34 cm^2^) is significantly different from the lesion area of transgenic lines T2-3 and T2-5 (only 0.19 and 0.16 cm^2^, respectively) (Fig 8B)These results indicate that the overexpression of the *GmPRP* gene in tobacco and soybean plants improved the resistance to *P*. *nicotianae* and *P*. *sojae*, respectively.

## Discussion and Conclusions

In the interaction between soybean and *P*. *sojae*, certain plant PR proteins are accumulated upon *P*. *sojae* infection, and these proteins may be associated with *P*. *sojae* resistance. Moy et al. (2004) [68] reported that PRa in a sensitive soybean was found to be upregulated at 3 h after infection with *P*. *sojae* and to maintain active expression until the last sampling time (48 h). Narayanan et al. (2009) [69] found two defense-related *PR* genes that were upregulated in resistant soybean upon infection by *P*. *sojae*. Xu et al. (2012) [56] reported microarray analysis showing four PR protein genes that were upregulated during infection by *P*. *sojae* and then confirmed that result using real-time PCR. Among these PR proteins, *GmPR10* protein was demonstrated to play an important role in the host defense against *P*. *sojae* infection [50].

In a previous study, a novel upregulated cDNA encoding a PRP was screened in the highly resistant soybean cultivar ‘Suinong 10’ [56]. Here, the novel *PRP* gene (termed *GmPRP*) and corresponding gene products from soybean (*Glycine max*) were isolated and characterized to better understand the function of this protein in the defense against *P*. *sojae*. To the best of our knowledge, this study is the first report on the biological activity of GmPRP protein from soybean in the defense against a pathogen. Sequence analysis indicating that GmPRP contained no signal peptide suggested that it may be an intracellular protein located in the cell membrane and cytoplasm, and this was verified with subcellular localization of the GmPRP protein in Arabidopsis protoplast cells (Fig 4). Subcellular localization of the GmPRP protein was similar to other intracellular PR proteins, it may be secreted into cell plasma membrane to resist pathogen after being made in the cytoplasm [70]. Most of the intracellular PR genes possess introns and exons, and *GmPRP* also contained introns and exons. The prediction of the three-dimensional (3D) structure of GmPRP (Fig 1B) was very similar to those of certain other PR proteins, including GmPR10 [50]. However, the predicted GmPRP structure contained a particularly conserved NTF2-like motif that belongs to the Nuclear transport factor 2 (NTF2) superfamily (Fig 1C). This family includes members of the NTF2 family, Delta-5-3-ketosteroid isomerases, scytalone dehydratases, and the beta subunit of Ring hydroxylating dioxygenases [71]. Some reports have provided direct evidence that NTF2 is required for the nuclear import of RanGDP, which is associated with cell proliferation and gene regulation [72, 73]. Hence, how GmPRP protein mediate NTF2 domain against *P*. *sojae* still need further study in the plant defense.

To further understand the function of *PR* genes in plant defense reactions, the expression patterns of *PR* genes was analyzed by various stimulus [74]. The data presented in this paper demonstrate that the expression of the *GmPRP* gene could be strongly induced by JA, SA, ABA and ET. Similar results have previously been reported in other plants in which PR proteins were induced by various treatments [31–35]. These results suggest that the expression of *GmPRP* may depend on the SA, JA, ABA and ET signal transduction pathways. Some reports have shown that the *PR* genes could be induced by certain factors in response to ET treatment, such as ethylene-response factors (ERFs) [75], which contain a conserved AP2/ERF domain that binds to the GCC box elements present in the promoters of PR genes [76]. However, no GCC box was found in the promoter of *GmPRP* (S2 Fig), suggesting that it may not be the direct target of ERFs.

*P*. *sojae* is a soil-borne pathogen that can survive for many years in soil and depends on sporangia and zoospore formation, as well as the direct penetration of hyphae between the cell walls of the epidermis, to infect soybean [55, 77]. The inhibition of sporangia and zoospore formation or hyphal development will be useful for the host’s resistance to *P*. *sojae*. In the present study, recombinant GmPRP protein significantly inhibited the hyphae growth of *P*. *sojae in vitro* (Fig 5A), but whether this protein inhibits hyphal development *in vivo* requires further research.

In the RNA degradation assay, the recombinant GmPRP protein showed ribonucleolytic activity, where part of the RNA was degraded within 2 h of incubation (Fig 5B), indicating that ribonucleolytic activity may be one of the important roles of this protein in the plant defense response to pathogen attack. Furthermore, the increased expression of *GmPRP* in transgenic tobacco and soybean plants may contribute to enhanced resistance against pathogens. In further experiments, *GmPRP* was successfully transformed into tobacco and soybean plants, and the antimicrobial activities of *GmPRP* were evaluated through the inoculation of transgenic tobacco and soybean plants overexpressing *GmPRP*. These transgenic tobacco and soybean plants showed enhanced levels of resistance to *P*. *nicotianae* and *P*. *sojae*, respectively. These results suggest that the enhanced resistance to pathogens in tobacco and soybean plants may be associated with the overexpression of *GmPRP*.

In conclusion, we characterized a novel soybean *PRP* gene from ‘Suinong 10’ soybean after inoculation with *P*. *sojae*. To the best of our knowledge, this is the first report of a *PRP* gene from soybean to describe its functional accreditation in imparting defense against a pathogen. Further characterization of *GmPRP* and its regulation under ambient and stress environments will enhance our understanding of the molecular cross-talk among various signaling pathways mediating plant defense responses.

## Supporting Information

S1 Fig Expression and purification of GmPRP protein from E. coli BL21 (DE3). (A) Lanes 1–7, E. coli BL21 containing the pET-29b(+) vector harboring the *GmPRP* gene induced by IPTG for 2, 4, and 6 h, respectively; Lane 1, precipitate from E. coli BL21 transformed without pET-29b(+) upon induction by 0.5 mM IPTG; Lane 2, precipitate from E. coli BL21 transformed with pET-29b(+) without induction by 0.5 mM IPTG; Lane 3, precipitate from E. coli BL21 transformed with pET-29b(+) upon induction by 0.5 mM IPTG; Lane 4, precipitate from E. coli BL21 transformed with the recombinant *GmPRP* and pET-29b(+) without induction by 0.5 mM IPTG; Lanes 5–7, precipitates from E. coli BL21 transformed with the recombinant *GmPRP* and pET-29b(+) with induction by 0.5 mM IPTG for 2, 4, and 6 h, respectively. (B) Purification of recombinant GmPRP protein from E. coli BL21 transformed with the pET-29b(+) vector containing the *GmPRP*. Lane 8, purified recombinant GmPRP protein. Lane M, protein marker.
(TIF)

S2 Fig 1100 bp putative promoter sequences of Gm*PRP* gene upstream of ATG. The nucleotide position of the ATG translation initiation codon was assigned as position 1 in the nucleotide sequence, and the nucleotide positions upstream of position 1 were shown as minus numbers. The putative cis-acting elements were upperlined with a gray background, and the names were shown above the elements. Light responsive element: ACE, Box 4, Bow I, G-Box, G-box, Gap-box, Sp1, ATCT-motif, MRE. cis-acting regulatory element essential for the anaerobic induction: ARE; fungal elicitor responsive element: Box-W1; cis-acting element to heat stress responsiveness: HSE; cis-acting element involved in defense and stress the responsiveness: TC-rich repeats; cis-acting element in salicylic acid: TCA-element; auxin-responsive element: TGA-element; binding site of AT-rich DNA bingding protein (ATBP-1): AT-rich element; cis-acting regulatory element involved in MeJA-responsiveness: CGTCA-motif; gibberellins-responsive element: GARE-motif; cis-acting regulatory element required for endosperm expression: Skn-1 motif.
(TIF)
