# Supplementary figures and images for: Isolation and Characterization of a Novel Pathogenesis-Related Protein Gene (GmPRP) with Induced Expression in Soybean (Glycine max) during Infection with Phytophthora sojae
Source: PLoS One. 2015 Jun 26;10(6):e0129932. doi: 10.1371/journal.pone.0129932 (PMC4482714; doi:10.1371/journal.pone.0129932)

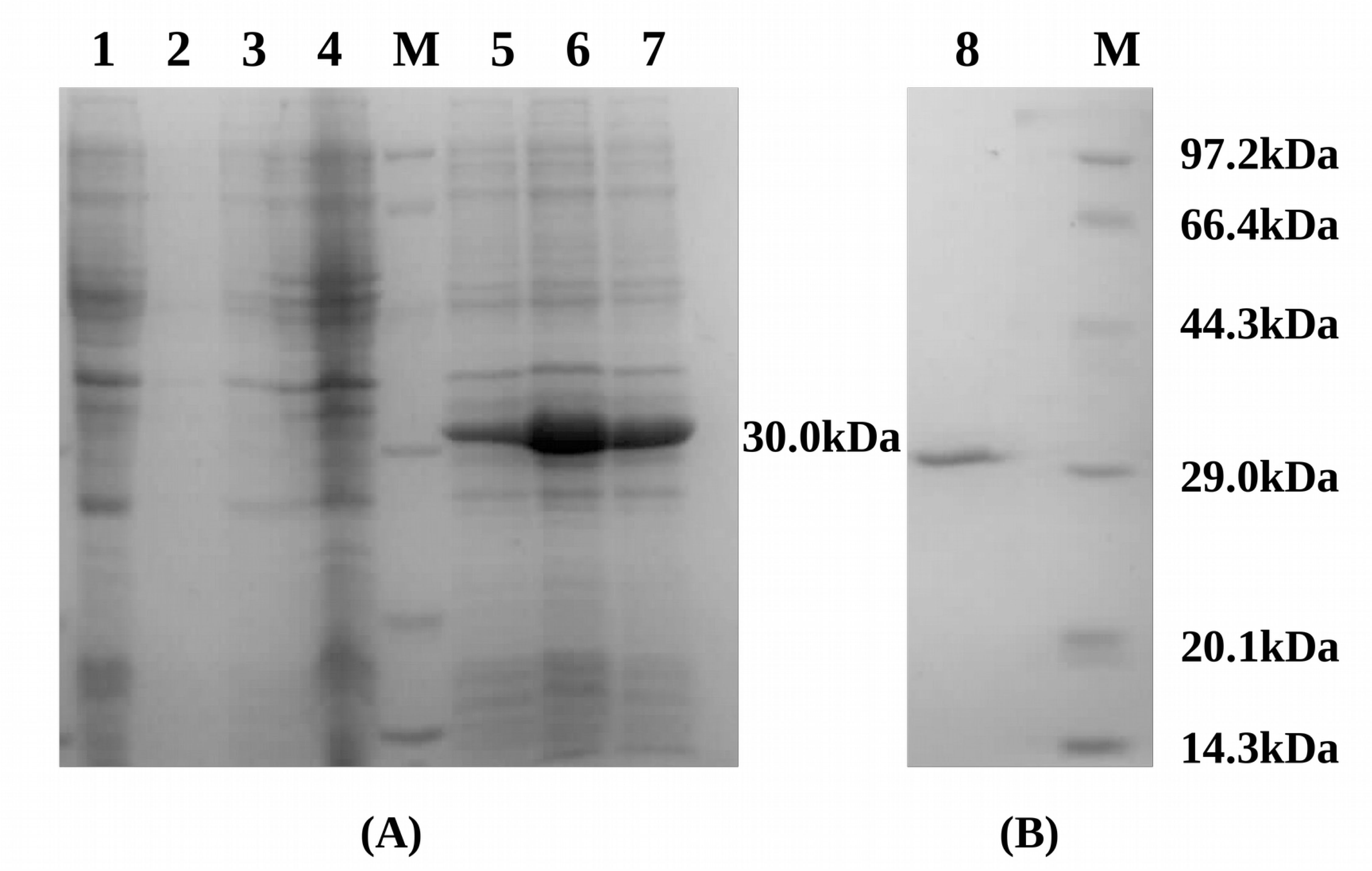

Supplement: S1 Fig — (A) Lanes 1–7, E. coli BL21 containing the pET-29b(+) vector harboring the GmPRP gene induced by IPTG for 2, 4, and 6 h, respectively; Lane 1, precipitate from E. coli BL21 transformed without pET-29b(+) upon induction by 0.5 mM IPTG; Lane 2, precipitate from E. coli BL21 transformed with pET-29b(+) without induction by 0.5 mM IPTG; Lane 3, precipitate from E. coli BL21 transformed with pET-29b(+) upon induction by 0.5 mM IPTG; Lane 4, precipitate from E. coli BL21 transformed with the recombinant GmPRP and pET-29b(+) without induction by 0.5 mM IPTG; Lanes 5–7, precipitates from E. coli BL21 transformed with the recombinant GmPRP and pET-29b(+) with induction by 0.5 mM IPTG for 2, 4, and 6 h, respectively. (B) Purification of recombinant GmPRP protein from E. coli BL21 transformed with the pET-29b(+) vector containing the GmPRP. Lane 8, purified recombinant GmPRP protein. Lane M, protein marker. (TIF) [file pone.0129932.s001.tif]

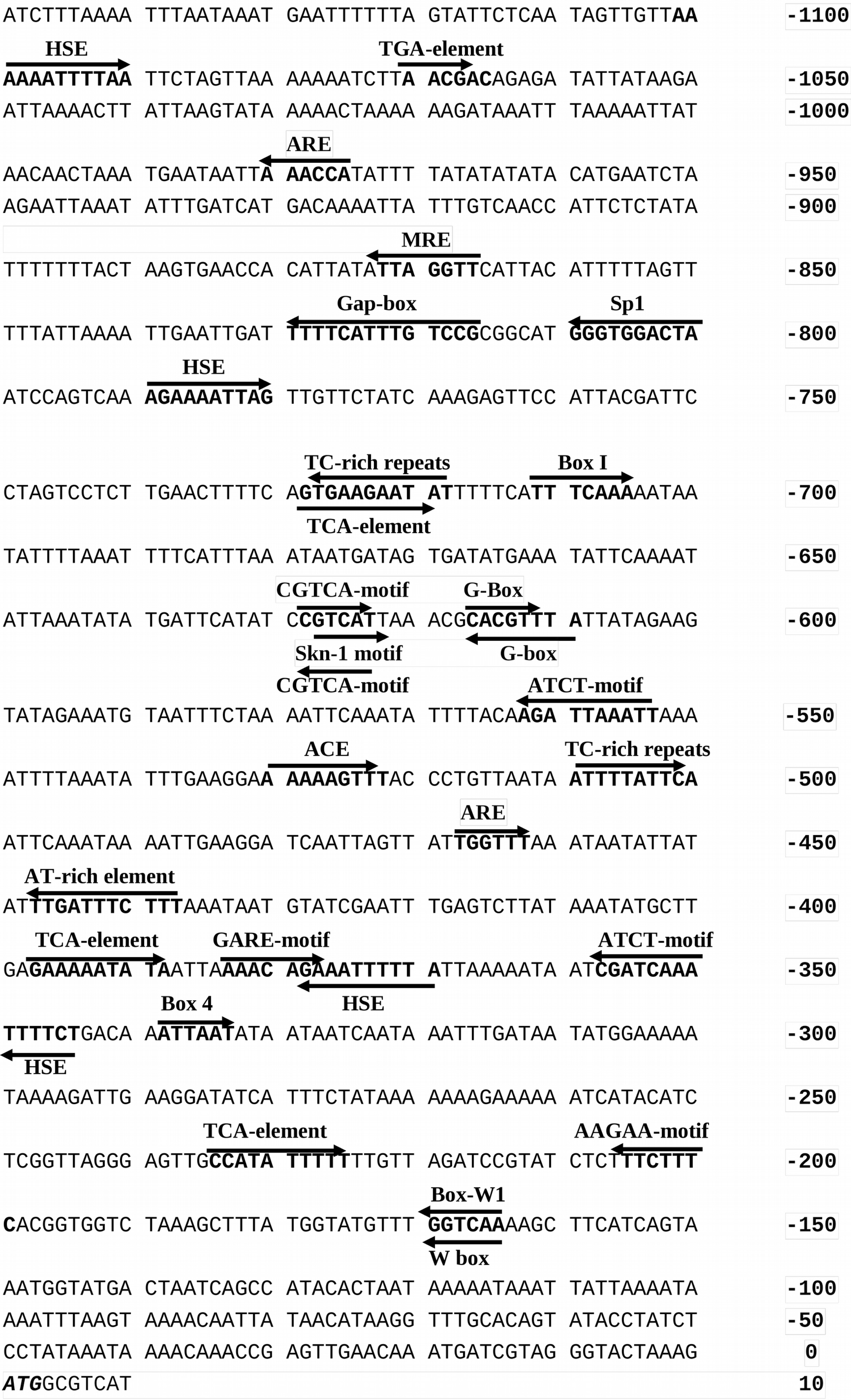

Supplement: S2 Fig — The nucleotide position of the ATG translation initiation codon was assigned as position 1 in the nucleotide sequence, and the nucleotide positions upstream of position 1 were shown as minus numbers. The putative cis-acting elements were upperlined with a gray background, and the names were shown above the elements. Light responsive element: ACE, Box 4, Bow I, G-Box, G-box, Gap-box, Sp1, ATCT-motif, MRE. cis-acting regulatory element essential for the anaerobic induction: ARE; fungal elicitor responsive element: Box-W1; cis-acting element to heat stress responsiveness: HSE; cis-acting element involved in defense and stress the responsiveness: TC-rich repeats; cis-acting element in salicylic acid: TCA-element; auxin-responsive element: TGA-element; binding site of AT-rich DNA bingding protein (ATBP-1): AT-rich element; cis-acting regulatory element involved in MeJA-responsiveness: CGTCA-motif; gibberellins-responsive element: GARE-motif; cis-acting regulatory element required for endosperm expression: Skn-1 motif. (TIF) [file pone.0129932.s002.tif]
